# Effects of alcohol drinking and smoking on pancreatic ductal adenocarcinoma mortality: A retrospective cohort study consisting of 1783 patients

**DOI:** 10.1038/s41598-017-08794-1

**Published:** 2017-08-29

**Authors:** Shuisheng Zhang, Chengfeng Wang, Huang Huang, Qinglong Jiang, Dongbing Zhao, Yantao Tian, Jie Ma, Wei Yuan, Yuemin Sun, Xu Che, Jianwei Zhang, Haibo Chen, Yajie Zhao, Yunmian Chu, Yawei Zhang, Yingtai Chen

**Affiliations:** 1 Department of Pancreatic and Gastric Surgery, National Cancer Center/Cancer Hospital, Chinese Academy of Medical Sciences and Peking Union Medical College, Beijing, 100021 China; 2 Department of Surgery, Yale School of Medicine, Yale Cancer Center, New Haven, CT USA; 3 State Key Laboratory of Molecular Oncology, National Cancer Center/Cancer Hospital, Chinese Academy of Medical Sciences and Peking Union Medical College, Beijing, 100021 China; 4 Clinical Immunology Center, Chinese Academy of Medical Science, Beijing, 100730 China; 5 Department of Cardiology, Second Affiliated Hospital, College of Medicine, Zhejiang University, Hangzhou, China

## Abstract

The effects of alcohol drinking and smoking on pancreatic ductal adenocarcinoma (PDAC) mortality are contradictory. Individuals who were diagnosed as PDAC and hospitalized at the China National Cancer Center between January 1999 and January 2016 were identified and included in the study. Ultimately, 1783 consecutive patients were included in the study. Patients were categorized as never, ex-drinkers/smokers or current drinkers/smokers. Hazard ratios (HRs) of all-cause mortality and 95% confidence intervals (CIs) were estimated using Cox proportional hazards models. Compared with never drinkers, the HRs were 1.25 for ever drinkers, 1.24 for current drinkers, and 1.33 for ex-drinkers (trend *P* = 0.031). Heavy drinking and smoking period of 30 or more years were positive prognostic factors for PDAC. For different smoking and alcohol drinking status, only subjects who are both current smokers and current drinkers (HR, 1.45; 95% CI, 1.03–2.05) were associated with reduced survival after PDAC compared to those who were never smokers and never drinkers. Patients who are alcohol drinkers and long-term smokers before diagnosis have a significantly higher risk of PDAC mortality. Compared to those who neither smoker nor drink, only patients who both smokers and drinkers were associated with reduced survival from PDAC.

## Introduction

Pancreatic ductal adenocarcinoma (PDAC) is a leading cause of cancer mortality worldwide with over 330,000 new cases and approximately the same number of deaths annually^1–4^. Patient survival is greatly influenced by disease stage at presentation, but few other markers of survival have been well characterized.

Chronic alcohol intake can cause structural and functional impairment in the pancreas. These changes lead to advanced contact between cathepsin B (lysosomal enzyme that activates trypsinogen) and digestive enzymes, ultimately resulting in premature intracellular activation of digestive enzymes and autodigestive injury to the pancreas^5^. So far, data that elucidate the prognostic role of alcohol in patients with PDAC have been limited; moreover, they are contradictory^6–10^. Alcohol drinking was reported to decrease survival in patients with PDAC in some studies^6, 9^. However, several studies^7, 8, 10^ did not confirm this finding.

Cigarette smoke contains a complex mixture of over 4000 compounds that have carcinogenic effects, which influences all aspects of tumor biology including initiation, progression and metastasis through mutations, inflammation and immunosuppression^11^. Smoke exposure can increase the expression of heparin-binding epidermal growth factor-like growth factor (HB-EGF), leading to a faster progression of PDAC^11^. Several studies^6, 10, 12–16^ investigated the effects of smoking on PDAC survival. Some studies^6, 10, 12, 15^ have reported a positive association between smoking and overall survival from PDAC. On the other hand, other studies^13, 14, 16^ have found that smoking status was inversely associated with PDAC survival.

The aim of this study was to provide further information on the impact of smoking and alcohol consumption on overall survival after a diagnosis of PDAC, by analyzing data on a relatively large number of Chinese patients.

## Materials and Methods

A retrospective cohort study using prospectively collected data was conducted at the Cancer Hospital of the Chinese Academy of Medical Sciences, China National Cancer Center. Individuals who were diagnosed as PDAC and hospitalized between January 1999 and January 2016 were identified and included in the study. Histological or cytological confirmation was available for all the included patients. Histologically confirmed neuroendocrine tumors or other types of pancreatic tumors were excluded. All patients had no previous diagnosis of any cancer. The study protocols were approved by the Institutional Review Board at the China National Cancer Center.

The following information were obtained by trained investigators from the medical records: sociodemographic characteristics, anthropometric measures (height and weight measured at diagnosis as well as usual adult weight reported by the patients), smoking (number of cigarettes per day (/d), duration of smoking, years since stopped smoking), and alcohol consumption (the volume of alcohol/d, duration of drinking, and, years since stopped drinking). Ex-drinkers were defined as subjects who had stopped drinking more than 1 year before cancer diagnosis. Ex-smokers were defined as those who had stopped smoking more than 1 year before cancer diagnosis. The amount of ethanol consumed daily was calculated in grams, and drinkers were categorized into three groups: light drinkers (<15.6 g/d), moderate drinkers (≥15.6 g/d to <53.5 g/d) and heavy drinkers (≥53.5 g/d). Smokers were categorized into two groups according to duration of smoking, long-term smokers (duration < 29 years) and short-term smokers (duration ≥ 29 years). Body mass index (BMI) was calculated according to Quetelet’s index (weight/height^2^, kg/m^2^). The BMI at diagnosis was calculated by the height and weight measured at diagnosis. The usual BMI was calculated by the height measured at diagnosis and self-reported usual adult weight, which was defined as the usual weight one year before the disease diagnosis. Information on clinical characteristics of pancreatic cancer (eg, tumor stage) was also recorded.

Vital status was ascertained using two different procedures. We obtained data from population registries from local authorities, municipal registration offices and local health units. Data were collected on date and place of information retrieval (if the subject was still alive), death, or migration outside of China. For patients whose information could not be ascertained through population registries, we obtained the vital status by calling the patients or next of kin. Information on cause(s) of death was not collected.

The end point was the death of patients or the last follow-up time (Marth 31, 2016). Eleven (0.6%) patients had missing information on date of diagnosis. 119 subjects (6.2%) were lost to follow-up or missed information on date of death Therefore a total of 1783 subjects (94.2%) were included in the final analysis. Overall, 143 patients were alive at the end of follow-up (maximum follow-up time of 17 years) and 1640 had died.

### Statistical Analysis

Overall survival was analyzed using Kaplan-Meier product-limit survival curve estimates and log-rank tests for comparison between groups. Overall survival was defined as the time from date of diagnosis of pancreatic cancer to date of end of follow-up or death. In the figures, we cut follow-up time at 5 years. We tested the proportional hazards assumption by including time-dependent effects in the model (ie, a covariate for interaction of the predictor and the logarithm of survival time), and no violation was found. Hazard ratios (HRs) of all-cause mortality and the corresponding 95% CIs were estimated using Cox proportional hazards models and included terms for age, calendar period at diagnosis, sex, BMI and tumor stage. Furthermore, in the analyses on smoking and alcohol consumption, the latter covariates were reciprocally adjusted. All tests were 2-sided, and P < 0.05 was taken as statistically significant. Statistical analyses were performed using SAS 9.1 (SAS Institute, Cary, NC) statistical software.

## Results

A total of 1,783 consecutive patients with data available were identified and included in the study. Their median survival was 8.26 months and their 5-year overall survival was 4.2%.

Table 1 shows the main characteristics of 1,783 patients with PDAC included in the study and the corresponding HRs and 95% CIs for all-cause mortality. Approximately two thirds of the patients were aged 50 to 69 years at diagnosis, with a mean age of 58.7 years, and about 56.6% of the cases were males. Mean usual BMI and BMI at diagnosis were 24.19 kg/m^2^ and 23.3 kg/m^2^ respectively. About half of the patients had a BMI of less than 23 kg/m^2^ at diagnosis, however, only a third of the patients had a usual BMI of less than 23 kg/m^2^. More than 70% of the patients were diagnosed as metastatic PDAC. The HR of the female patients for all-cause mortality was 1.16 (95% CI, 1.01–1.33) versus the male patients. As compared with the patients in the resectable stage, the HR was 1.22 (95% CI, 1.03–1.62) for those in the unresectable and locally advanced stage, and 1.17 (95% CI, 0.93–1.48) for those in the metastatic stage. No meaningful difference in survival after PDAC emerged according to age, BMI at diagnosis and usual BMI.

**Table 1:** Characteristics and overall survival (OS) of 1,783 patients with pancreatic cancer, 1999–2016.

Characteristic	No. of Cases (%)	Median OS, mo	HR (95%CI)
Age at diagnosis, y			
<50	355 (19.9%)	8.6 (7.4–10.4)	1 (Reference)
50–59	620 (34.8%)	9.4 (7.3–11.7)	0.99 (0.79–1.24)
60–69	547 (30.7%)	7.3 (6.2–8.6)	1.17 (0.93–1.48)
≥70	261 (14.6%)	7.8 (6.4–9.9)	1.14 (0.86–1.50)
Trend, χ^2^ (p value)			2.85 (0.092)
Gender			
Male	1,009 (56.6%)	9.0 (7.7–9.7)	1 (Reference)
Female	774 (43.4%)	8.2 (7.3–9.2)	1.16 (1.01–1.33)
BMI at diagnosis (kg/m^2^)			
<23	880 (49.3%)	8.5 (7.3–9.7)	1 (Reference)
23–27.49	741 (41.6%)	8.1 (7.2–9.5)	1.02 (0.86–1.21)
≥27.5	162 (9.1%)	6.1 (4.8–12.4)	1.15 (0.85–1.57)
Usual BMI (kg/m^2^)			
<23	686 (38.5%)	8.3 (7.3–9.9)	1 (Reference)
23–27.49	791 (44.4%)	8.1 (7.2–9.4)	0.95 (0.80–1.14)
≥27.5	306 (17.1%)	7.6 (6.0–10.2)	1.07 (0.85–1.35)
Stage group			
Resectable	217 (12.2%)	12.4 (8.7–15.0)	1 (Reference)
Unresectable & locally advanced	316 (17.7%)	9.0 (6.6–10.7)	1.22 (1.03–1.62)
Metastatic	1,250 (70.1%)	5.4 (4.7–6.1)	1.92 (1.61–2.33)

Table 2 shows the distribution of the pancreatic cancer cases and their HRs of all-cause mortality according to tobacco smoking and alcohol drinking.

**Table 2:** Alcohol drinking habits, smoking habits, and overall survival (OS) of 1,783 patients with pancreatic cancer.

Characteristic	No. of Cases (%)	Median OS, mo	HR (95%CI)
Alcohol drinking			
Never drinkers	1,377 (77.2%)	8.8 (7.8–9.8)	1 (Reference)
Drinkers	398 (22.3%)	6.5 (5.7–7.5)	1.25 (1.02–1.54)
Current drinkers	318 (17.8%)	6.5 (5.7–7.7)	1.24 (1.00–1.54)
Ex-drinkers	80 (4.5%)	6.3 (3.2–9.0)	1.33 (0.88–2.00)
Trend, χ^2^ (*p* value)			4.66 (0.031)
Amount of alcohol consumption			
Light drinkers	28 (1.6%)	7.6 (5.5–16.3)	1.06 (0.12–1.91)
Moderate drinkers	126 (7.1%)	6.9 (5.7–13.9)	1.10 (0.83–1.44)
Heavy drinkers	232 (13.0%)	6.4 (3.0–7.5)	1.45 (1.03–2.05)
rend, χ^2^ (*p* value)			2.51 (0.11)
Tobacco smoking			
Never smokers	1,243 (69.7%)	8.6 (7.4–9.6)	1 (Reference)
Smokers	527 (29.6%)	7.6 (6.9–9.0)	1.01 (0.85–1.20)
Current smokers	427 (23.9%)	7.9 (7.0–9.5)	1.02 (0.87–1.21)
Ex-smokers	100 (5.6%)	7.1 (4.8–9.0)	1.20 (0.85–1.68)
Trend, χ^2^ (*p* value)			0.28 (0.59)
Time since stopping			
Long-term (≥10y)	14 (0.8%)	7.6 (6.9–9.4)	1.54 (0.88–2.70)
Short-term (1–9y)	86 (4.8%)	7.7 (3.8–9.0)	1.29 (0.83–2.18)
Trend, χ^2^ (*p* value)			0.0070 (0.93)
No. of cigarettes/d			
1–19	306 (17.2%)	7.7 (6.6–9.4)	1.06 (0.85–1.32)
≥20	221 (12.4%)	7.3 (6.4–8.8)	1.94 (0.79–1.37)
Trend, χ^2^ (*p* value)			0.60 (0.44)
Duration of smoking			
1–29y	250 (14.0%)	8.4 (6.6–13.2)	1.04 (0.66–1.28)
≥30y	277 (15.5%)	6.5 (5.8–7.5)	1.40 (1.11–1.76)
Trend,χ^2^ (*p* value)			2.03 (0.15)
Smoking and alcohol drinking status			
Never smoker and never drinker	1,074 (60.2%)	8.6 (7.4–9.6)	1 (Reference)
Current smoker and never drinker	316 (17.7%)	8.2 (7.7–11.2)	1.02 (0.69–1.18)
Current drinker and never smoker	65 (3.6%)	7.0 (4.1–16.2)	1.15 (0.68–1.64)
Current smoker and current drinker	328 (18.4%)	6.4 (5.7–7.3)	1.26 (1.02–1.56)

**Figure 1:**
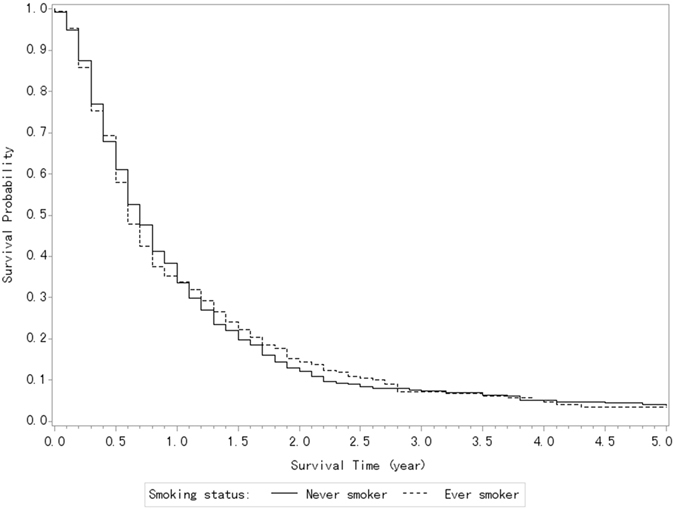
Kaplan-Meier Curves of the patients with pancreatic cancer according to their tobacco smoking status (log-rank test, P = 0.67).

67.7% of the cases had never smoked, 23.9% were current smokers, 5.6% were ex-smokers, and 2.8% had missing smoking information. The corresponding median overall survival was 8.6 months in the never smokers, 7.9 months in the current smokers, and 7.1 months in the ex-smokers. Figure 1 shows the survival curves of the patients with PDAC according to their tobacco smoking. The former smokers had an insignificantly different survival rate as compared to the never smokers (log-rank test *P* = 0.67).

The multivariate HR (Table 2) was 1.01 (95% CI, 0.85–1.20) for the former smokers, 1.02 (95% CI, 0.87–1.21) for the current smokers, and 1.20 (95% CI, 0.85–1.68) for the ex-smokers versus the never smokers (P for trend = 0.59). With reference to the subjects who had stopped smoking—as compared with the never smokers—their HR was 1.54 (95% CI, 0.88–2.70) for the long-term ex-smokers (i.e., those who stopped smoking since > = 10 years ago) and 1.29 (95% CI, 0.83–2.18) for the short-term ex-smokers (P for trend = 0.93).

Increasing amount and duration of smoking had unfavorable effects on overall survival after pancreatic cancer, since the HR of death was 1.06 (95% CI, 0.85–1.32) for smoking up to 19 cigarettes/d and 1.94 (95% CI, 0.79–1.37) for 20 or more (P for trend = 0.44), and 1.04 (95% CI, 0.66–1.28) for a duration of smoking less than 30 years and 1.40 (95% CI, 1.11–1.76) for 30 or more years, as compared with the never smokers (P for trend = 0.15).

**Figure 2:**
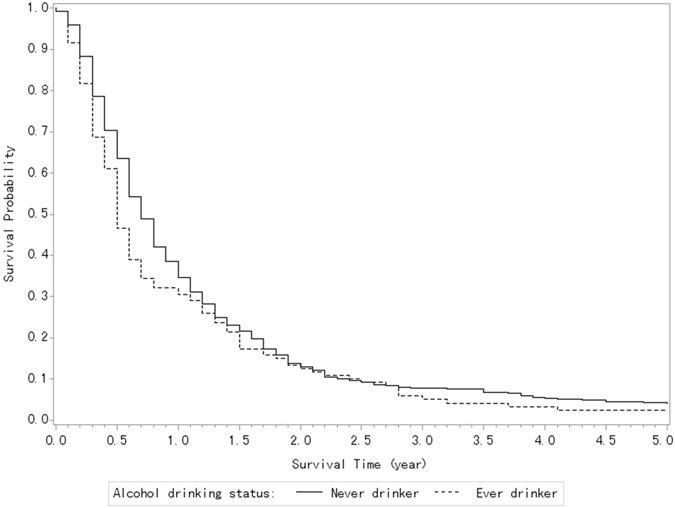
Kaplan-Meier Curves of the patients with pancreatic cancer according to their alcohol drinking status (log-rank test, P = 0.074).

About 77.2% of the patients were never drinkers, 17.8% were current drinkers, 4.5% were ex-drinkers, and 0.5% had missing information on alcohol consumption. Figure 2 shows the survival curves of the patients with pancreatic cancer according to alcohol consumption. No meaningful difference in overall survival was reported among the never drinkers, current drinkers, and ex-drinkers, with a log-rank test *P* = 0.074. Compared with the never drinkers, the multivariate HR (Table 2) was 1.25 (95% CI, 1.02–1.54) for the former drinkers, 1.24 (95% CI, 1.00–1.54) for the current drinkers, and 1.33 (95% CI, 0.88–2.00) for the ex-drinkers (P for trend = 0.031). As for the association between the amount of alcohol consumption and survival, only heavy drinkers were associated with reduced survival after PDAC as compared with the never drinkers (HR, 1.45; 95% CI, 1.03–2.05).

**Figure 3:**
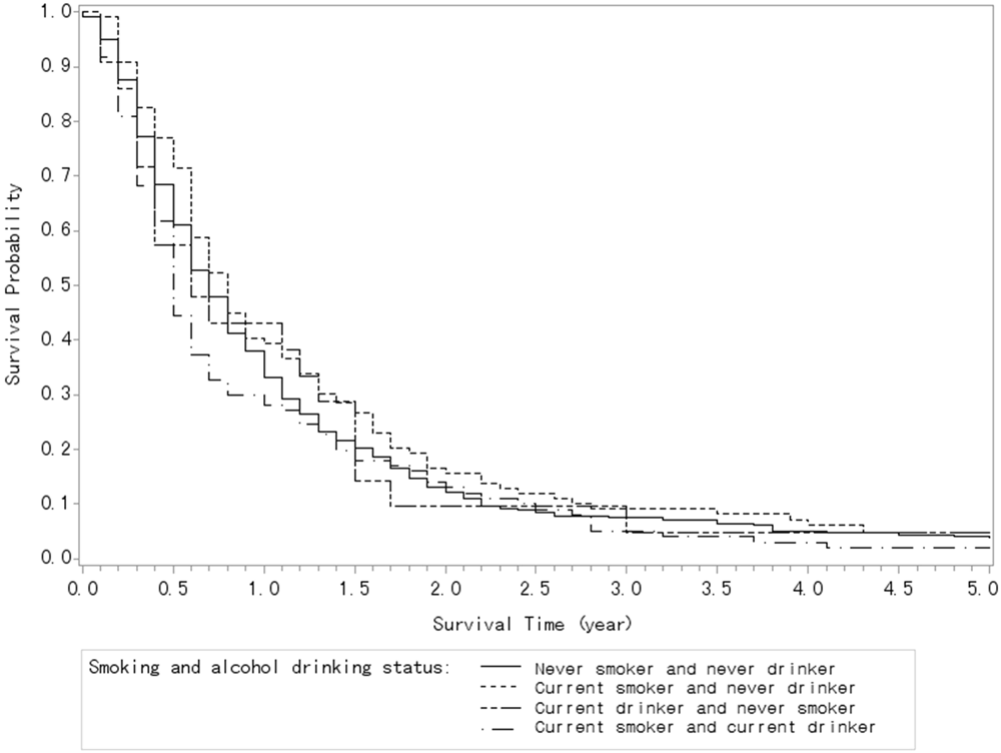
Kaplan-Meier curves of the patients with pancreatic cancer according to their smoking and alcohol drinking status (log-rank test, P = 0.10).

For different smoking and alcohol drinking statuses, only subjects who were both current smokers and drinkers (HR, 1.45; 95% CI, 1.03–2.05) were associated with reduced survival after PDAC as compared to those who were never smokers and never drinkers. Although the interaction between smoking and alcohol intake on survival was not statistically significant (*P* = 0.24). Figure 3 shows the survival curves of the patients with pancreatic cancer according to smoking and alcohol drinking status (log-rank test, *P* = 0.10).

### Subgroup Analysis

After stratification by clinical stage, the HRs of all-cause mortality were also reported according to tobacco smoking and alcohol drinking. For the 217 pancreatic cancer patients who underwent pancreatectomy, a smoking period of 30 or more years (versus never smokers; HR, 1.90; 95% CI, 1.26–3.35) and a status of being both current smoker and current drinker (versus patients who were never smoker and never drinker; HR, 1.91; 95% CI, 1.01–4.35) were positive factors for PDAC mortality (Supplemental Table 1).

For the 316 patients with unresectable and locally advanced pancreatic cancer, smoking 20 or more cigarettes/d (versus never smokers; HR, 2.10; 95% CI, 1.09–4.02) was the sole positive factor for PDAC mortality (Supplemental Table 2).

For the 1,250 patients with distant metastatic pancreatic cancer, only heavy drinkers were associated with reduced survival from PDAC as compared to the never drinkers (HR, 1.55; 95% CI, 1.01–2.38) (Supplemental Table 3).

## Discussion

The findings of this study confirmed a decreased survival rate of pancreatic cancer patients with alcohol drinking and long-term tobacco smoking.

From this investigation, we found a positive association between alcohol consumption and survival. When the amount of alcohol consumption was taken into consideration, only heavy drinkers were associated with a decreased survival rate. Light and moderate drinkers did not show the same result.

In contrast, many previous studies have expressed no association between alcohol intake and pancreatic cancer mortality^7, 8, 10, 17^. In one of the largest cohort studies published to date^7^, which included 3,012 pancreatic cancer cases, the risk ratio (RR) of pancreatic cancer death related to the consumption of at least 1 drink per day was 1.0 (95% CI, 0.9–1.1) for men and 1.0 (95% CI, 0.8–1.1) for women.

Two other investigators^6, 9^, however, got opposite results. In a retrospective case-control study^6^ involved 248 pancreatic cancer cases, the RR of pancreatic cancer mortality associated with consumption of alcohol was 1.83 (95% CI, 1.1–3.0) based on 163 cases. Another cohort study^9^ reported significantly higher risks of pancreatic cancer mortality associated with the consumption of 3 drinks/d (RR, 1.25; 95% CI, 1.11–1.42) or at least 4 drinks/d (RR, 1.25; 95% CI, 1.11–1.42), while no association was found between pancreatic cancer mortality and “moderate” alcohol consumption (i.e., <3 drinks per day)^9^.

Several factors might be responsible for this phenomenon, although its underlying mechanism is currently unclear. The biological mechanism for the association between alcohol consumption and pancreatic cancer is not fully understood, but it is well recognized that long-term heavy intake causes chronic alcoholic pancreatitis^18^, an established risk factor for pancreatic cancer. In addition, ethanol is metabolized in the oxidative and non-oxidative manners^19^ in the pancreas. Go *et al*.^20^ hypothesized several mechanisms by which metabolites might affect inflammation and carcinogenesis, including activation of nuclear transcription factors, increased production of reactive oxygen species, and dysregulation of cell proliferation and apoptosis. Chronic alcohol intake can cause structural and functional impairment of the pancreas. These changes lead to advanced contact between cathepsin B, i.e., lysosomal enzyme that activates trypsinogen, and digestive enzymes, ultimately resulting in premature intracellular activation of digestive enzymes and autodigestive injury to the pancreas^5^.

Our study showed no meaningful difference between ever smoking and never smoking in terms of the prognosis of pancreatic cancer. But when taking the duration of smoking into consideration, we found a decreased survival rate among long-term smokers.

After stratification by clinical stage, positive factors for patients who underwent pancreatectomy included smoking period of 30 or more years, and smoking 20 or more cigarettes/d for patients with unresectable locally advanced PDAC.

A detrimental effect of smoking on survival has been previously reported for many kinds of cancer^6, 17, 21, 22^, especially those etiologically associated with tobacco, such as lung cancer^6^. The causal association between tobacco smoking and pancreatic cancer has been well established^23, 24^. However, few data are available on prediagnostic tobacco smoking as a determinant of survival after pancreatic cancer, with 7 studies showing significant critical roles of tobacco^6–8, 10, 12, 15, 25^, and 4 showing no association^13, 14, 16, 17^. Of course, only 1 study^10^ considered the effects of the amount and duration of smoking, except smoking status, and showed the poorest prognosis for current smokers in the categories of higher use, that is, 20 or more cigarettes/d and 30 or more years of smoking.

While the mechanisms underlying this phenomenon remained unclear, a possible explanation is that smokers might develop more aggressive tumors^17^. This is also supported by the prometastatic role of nicotine in pancreatic ductal adenocarcinoma according to its influence on the expression of matrix metalloproteinase 9 and the vascular endothelial growth factor^26^. In addition, the effect of smoking on survival requires a long time to accumulate.

In this study, there was a positive association between alcohol consumption and survival rate of the overall patients, but after stratification by smoking and alcohol drinking status, only patients who are both smokers and drinkers were associated with reduced survival from PDAC. The mechanisms underlying this phenomenon were unclear. A possible reason for this result is that the two factors can interact with each other and promote the development of pancreatic cancer.

Five-year overall survival rates reported in previous studies ranged from 3% to 8%^27, 28^. Our present study showed an overall survival rate of pancreatic cancer patients of 4.2% at 5 years since diagnosis, which was within the range of previous studies. The extremely low 5-year survival rate was mainly due to the fact that a large proportion of pancreatic cancer patients had metastatic diseases at diagnosis. Additionally, the lower rate in our study might also be at least in part explained by the fact that more advanced cases were more likely to be referred to the China National Cancer for treatment.

We failed to find the association between age at diagnosis and mortality from pancreatic cancer. Women have a survival advantage over men for most cancer sites, including the pancreas^29^. However, we reported a decreased survival rate of female patients from pancreatic cancer (HR, 1.16; 95% CI, 1.01–1.33) compared to male patients. Age or sex may not be the substantial factor that affects survival, and these effects may vary among different studies^16, 29–32^. Our study suggested no significant increase in all-cause mortality for overweight and obese patients, contrary to most^16, 17, 30, 33^–but not all^34, 35^–earlier investigations. Previous reports with positive results^16, 17, 30, 33^ always contributed the association to two points. First, a high BMI at diagnosis could be associated with an increased frequency of perioperative and postoperative complications from pancreatic resection^36^ or with a lower frequency of surgery, leading to increased morbidity and mortality. However, a previous meta-analysis reported no difference in the length of hospital stay, hospital mortality, and other major outcomes between normal-weight and overweight/obese patients^37^. Second, insulin resistance and hyperinsulinemia could be used to explain the reduced survival of overweight/obese pancreatic cancer patients, but no association between diabetes and overall survival was reported in this meta-analysis^38^. These two points of supporting the positive association between BMI and survival are untenable.

The major strengths of this study include its size, availability of detailed information about alcohol consumption and smoking, and availability of other data such as clinical stages, allowed for an analysis over a wide range of PDAC patients with stratification by smoking and alcohol drinking status, and clinical stages^9^. Despite these strengths, some study limitations should be recognized^9^. First, dietary information and past medical conditions (such as diabetes and chronic pancreatitis), which could affect the mortality of pancreatic cancer patients, were not considered here due to the lack of information. Second, there could be potential bias due to incomplete exposure ascertainment. However, we had only 0.5% participants missing alcohol information and 2.8% missing smoking information, suggesting the bias caused by missing information was unlikely. Third, we did not have biochemical data to evaluate their impacts. Fourth, there was no information on more detailed pathology or grading of pancreatic cancer. Finally, the absence of duration of alcohol drinking for ever drinkers and time since stopped for ex-drinkers limited the opportunity for more detailed information on the association between drinking and pancreatic cancer mortality^14^.

To sum up, patients who are alcohol drinkers and long-term smokers before diagnosis have a significantly higher risk of PDAC mortality. Compared to those who are neither smokers nor drinkers, only patients are both smokers and drinkers expressed reduced survival from PDAC.

## Electronic supplementary material

Supplemental material
