# Supplementary material for: Effects of alcohol drinking and smoking on pancreatic ductal adenocarcinoma mortality: A retrospective cohort study consisting of 1783 patients
Source: Sci Rep. 2017 Aug 29;7:9572. doi: 10.1038/s41598-017-08794-1 (PMC5574975; doi:10.1038/s41598-017-08794-1)
Supplement: Supplementary file 1 — Supplemental material [file 41598_2017_8794_MOESM1_ESM.docx]

**Effects of alcohol drinking and smoking on pancreatic ductal adenocarcinoma mortality: A retrospective cohort study consisting of 1783 patients**

**Authors:**

Shuisheng Zhang^1,*^, Chengfeng Wang^1,*^, Huang Huang^2,*^, Qinglong Jiang^1^, Dongbing Zhao^1^, Yantao Tian^1^, Jie Ma^3,4^, Wei Yuan^3,4^, Yuemin Sun^1^, Xu Che^1^, Jianwei Zhang^1^, Haibo Chen^5^, Yajie Zhao^1^, Yunmian Chu^1^, Yawei Zhang^2^, Yingtai Chen^1#^

^*^Authors made the same contributions to the project.

^#^Correspondence: Yingtai Chen, National Cancer Center/Cancer Hospital, Chinese Academy of Medical Sciences and Peking Union Medical College, 17 Panjiayuan Nanli, Beijing 100021, China (e-mail: yingtai.chen@yale.edu)

**Affiliations and postal addresses:**

^1^ Department of Pancreatic and Gastric Surgery, National Cancer Center/Cancer Hospital, Chinese Academy of Medical Sciences, Peking Union Medical College, Beijing 100021, China

^2^ Department of Surgery, Yale School of Medicine, Yale Cancer Center, New Haven, CT

^3^ State Key Laboratory of Molecular Oncology, National Cancer Center/Cancer Hospital, Chinese Academy of Medical Sciences, Peking Union Medical College, Beijing 100021, China

^4^ Clinical Immunology Center, Chinese Academy of Medical Science, Beijing, 100730, China

^5^ Department of Cardiology, Second Affiliated Hospital, College of Medicine, Zhejiang University, Hangzhou, China

**Supplemental Table-1** Alcohol drinking habits, smoking habits, and overall survival (OS) of 217 pancreatic cancer patients who underwent pancreatectomy

| **Characteristic** | | | **No. of Cases (%)** | **Median OS, mo** | **HR (95%CI)** |
| --- | --- | --- | --- | --- | --- |
| Alcohol drinking | | |  |  |  |
|  | Never drinker | | 166 (76.5%) | 12.4 (10.4-16.1) | 1 (Reference) |
|  | Former drinker | | 51 (23.5%) | 10.6 (8.5-13.9) | 1.29 (0.68-2.45) |
|  |  | Current drinker | 44 (20.3%) | 12.5 (9.5-17.7) | 1.24 (0.64-2.40) |
|  |  | Ex-drinker | 7 (3.2%) | 11.4 (8.5-16.2) | 1.32 (0.45-10.94) |
|  |  | Trend, χ^2^ (*p* value) |  |  | 0.96 (0.33) |
|  | Amount of alcohol consumption | |  |  |  |
|  |  | Light drinker | 5 (2.3%) | 11.1 (5.5-15.3) | 1.34 (0.11-2.33) |
|  |  | Moderate drinker | 22 (10.1%) | 10.0 (4.3-13.1) | 1.64 (0.47-2.75) |
|  |  | Heavy drinker | 22 (10.1%) | 10.3 (5.1-13.7) | 1.46 (0.90-2.73) |
|  |  | Trend, χ^2^ (*p* value) |  |  | 1.22 (0.27) |
| Tobacco smoking | | |  |  |  |
|  | Never smoker | | 129 (59.4%) | 13.4 (10.0-16.1) | 1 (Reference) |
|  | Former smoker | | 88 (40.6%) | 9.4 (4.5-16.7) | 1.29 (0.75-2.22) |
|  |  | Current smoker | 68 (31.3%) | 10.5 (3.7-24.2) | 1.31 (0.72-2.37) |
|  |  | Ex-smoker | 20 (9.2%) | 9.2 (5.5-34.6) | 1.23 (0.46-3.29) |
|  |  | Trend, χ^2^ (*p* value) |  |  | 0.64 (0.42) |
|  | Time since stopping | |  |  |  |
|  |  | Long-term (≥15 y) | 4 (1.8%) | 9.3 (6.5-16.0) | 1.99 (0.57-6.91) |
|  |  | Short-term (1-14 y) | 16 (7.4%) | 9.9 (5.5-19.5) | 1.21 (0.68-2.15) |
|  |  | Trend, χ^2^ (*p* value) |  |  | 0.57 (0.45) |
|  | No. of cigarettes/d | |  |  |  |
|  |  | 1-19 | 31 (14.3%) | 10.5 (6.5-19.5) | 1.40 (0.64-3.06) |
|  |  | ≥20 | 57 (26.3%) | 8.3 (3.3-17.8) | 1.91 (0.78-4.65) |
|  |  | Trend, χ^2^ (*p* value) |  |  | 0.12 (0.73) |
|  | Duration of smoking | |  |  |  |
|  |  | 1-29y | 44 (20.4%) | 14.5 (2.5-22.1) | 0.90 (0.40-2.05) |
|  |  | ≥30y | 44 (20.4%) | 9.5 (5.3-12.9) | 1.90 (1.26-3.35) |
|  |  | Trend, χ^2^ (*p* value) |  |  | 4.66 (0.031) |
| Smoking and alcohol drinking status | | |  |  |  |
|  | Never smoker and never drinker | | 118 (54.4%) | 18.7 (10.6-25.3) | 1 (Reference) |
|  | Current smoker and never drinker | | 47 (21.7%) | 13.3 (10.0-15.0) | 1.47 (0.94-2.14) |
|  | Current drinker and never smoker | | 11 (5.1%) | 16.4 (8.0-26.4) | 1.08 (0.33-1.91) |
|  | Current smoker and current drinker | | 41 (18.9%) | 9.3 (7.5-14.0) | 1.91 (1.01-4.35) |

**Supplemental Table-2** Alcohol drinking habits, smoking habits, and overall survival (OS) of 316 patients with unresectable and locally advanced pancreatic cancer

| **Characteristic** | | | **No. of Cases (%)** | **Median OS, mo** | **HR (95%CI)** |
| --- | --- | --- | --- | --- | --- |
| Alcohol drinking | | |  |  |  |
|  | Never drinker | | 275 (87.0%) | 10.0 (7.6-11.7) | 1 (Reference) |
|  | Former drinker | | 41 (13.0%) | 7.2 (4.8-18.0) | 1.17 (0.62-2.21) |
|  |  | Current drinker | 38 (12.2%) | 7.1 (2.9-14.6) | 1.06 (0.55-2.03) |
|  |  | Ex-drinker | 3 (0.8%) | 9.0 (5.3-24.1) | 1.11(0.71-7.61) |
|  |  | Trend, χ^2^ (*p* value) |  |  | 0.65 (0.42) |
|  | Amount of alcohol consumption | |  |  |  |
|  |  | Light drinker | 3 (0.0%) | 8.6 (4.3-11.1) | 1.02 (0.11-1.87) |
|  |  | Moderate drinker | 18 (5.3%) | 7.2 (2.0-20.1) | 0.94 (0.38-2.36) |
|  |  | Heavy drinker | 19 (6.1%) | 9.4 (3.6-17.2) | 1.59 (0.70-3.60) |
|  |  | Trend, χ^2^ (*p* value) |  |  | 1.00 (0.32) |
| Tobacco smoking | | |  |  |  |
|  | Never smoker | | 217 (68.7%) | 10.0 (7.5-12.0) | 1 (Reference) |
|  | Former smoker | | 99 (31.3%) | 9.4 (7.2-13.9) | 0.95 (0.63-1.43) |
|  |  | Current smoker | 85 (26.9%) | 9.7 (6.7-13.9) | 0.97 (0.64-1.48) |
|  |  | Ex-smoker | 14 (4.4%) | 9.0 (5.8-12.7) | 0.72 (0.22-2.37) |
|  |  | Trend, χ^2^ (*p* value) |  |  | 0.16 (0.69) |
|  | Time since stopping | |  |  |  |
|  |  | Long-term (≥15 y) | 2 (0.6%) | 9.0 (7.9-10.0) | 1.54 (0.21-11.49) |
|  |  | Short-term (1-14 y) | 12 (3.8%) | 9.7 (7.2-13.9) | 0.94 (0.62-1.42) |
|  |  | Trend, χ^2^ (*p* value) |  |  | 0.089 (0.77) |
|  | No. of cigarettes/d | |  |  |  |
|  |  | 1-19 | 37 (11.7%) | 9.2 (6.3-13.0) | 1.15 (0.66-1.60) |
|  |  | ≥20 | 62 (19.6%) | 7.4 (4.2-9.4) | 2.10 (1.09-4.02) |
|  |  | Trend, χ^2^ (*p* value) |  |  | 0.056 (0.81) |
|  | Duration of smoking | |  |  |  |
|  |  | 1-29y | 49 (15.5%) | 9.2 (6.3-17.2) | 0.89 (0.48-1.65) |
|  |  | ≥30y | 50 (15.8%) | 7.5 (4.2-13.9) | 1.39 (0.80-2.42) |
|  |  | Trend, χ^2^ (*p* value) |  |  | 2.88 (0.090) |
| Smoking and alcohol drinking status | | |  |  |  |
|  | Never smoker and never drinker | | 210 (66.5%) | 10.0 (7.5-12.1) | 1 (Reference) |
|  | Current smoker and never drinker | | 65 (20.6%) | 9.4 (7.3-13.9) | 0.96 (0.61-1.52) |
|  | Current drinker and never smoker | | 7 (2.2%) | 7.4 (4.8-20.2) | 1.26 (0.69-7.43) |
|  | Current smoker and current drinker | | 34 (10.7%) | 7.6 (3.1-16.0) | 1.12 (0.49-2.04) |

**Supplemental Table-3** Alcohol drinking habits, smoking habits, and overall survival (OS) of 1,250 patients with distant metastatic pancreatic cancer

| **Characteristic** | | | **No. of Cases (%)** | **Median OS, mo** | **HR (95%CI)** |
| --- | --- | --- | --- | --- | --- |
| Alcohol drinking | | |  |  |  |
|  | Never drinker | | 936 (74.9%) | 5.8 (4.9-6.6) | 1 (Reference) |
|  | Former drinker | | 306 (24.5%) | 4.5 (3.7-5.6) | 1.25 (0.99-1.59) |
|  |  | Current drinker | 236 (18.9%) | 4.5 (3.7-5.7) | 1.27 (0.98-1.64) |
|  |  | Ex-drinker | 70 (5.6%) | 4.4 (2.2-7.4) | 1.20 (0.77-1.86) |
|  |  | Trend, χ^2^ (*p* value) |  |  | 2.63 (0.10) |
|  | Amount of alcohol consumption | |  |  |  |
|  |  | Light drinker | 20 (1.6%) | 5.6 (4.3-14.6) | 1.05 (0.73-4.30) |
|  |  | Moderate drinker | 86 (6.9%) | 5.4 (3.9-12.2) | 1.12 (0.76-1.38) |
|  |  | Heavy drinker | 191 (15.3%) | 4.4 (1.2-5.5) | 1.55 (1.01-2.38) |
|  |  | Trend,χ^2^ (*p* value) |  |  | 0.65 (0.42) |
| Tobacco smoking | | |  |  |  |
|  | Never smoker | | 897 (71.8%) | 5.4 (4.5-6.6) | 1 (Reference) |
|  | Former smoker | | 340 (27.2%) | 5.3 (4.4-6.6) | 1.01 (0.82-1.26) |
|  |  | Current smoker | 274 (21.9%) | 5.4 (4.5-6.8) | 0.96 (0.76-1.21) |
|  |  | Ex-smoker | 66 (5.3%) | 4.4 (2.5-7.4) | 1.36 (0.91-2.02) |
|  |  | Trend, χ^2^ (*p* value) |  |  | 0.57 (0.45) |
|  | Time since stopping | |  |  |  |
|  |  | Long-term (≥15 y) | 8 (0.6%) | 5.7 (0.9-12.3) | 1.52 (0.77-3.02) |
|  |  | Short-term (1-14 y) | 58 (4.6%) | 5.3 (4.4-6.8) | 1.00 (0.80-1.24) |
|  |  | Trend, χ^2^ (*p* value) |  |  | 0.0015 (0.97) |
|  | No. of cigarettes/d | |  |  |  |
|  |  | 1-19 | 125 (10.0%) | 6.0 (4.6-7.6) | 0.87 (0.62-1.21) |
|  |  | ≥20 | 215 (17.2%) | 4.7 (4.0-6.6) | 1.07 (0.83-1.38) |
|  |  | Trend, χ^2^ (*p* value) |  |  | 0.62 (0.43) |
|  | Duration of smoking | |  |  |  |
|  |  | 1-29y | 157 (12.6%) | 6.2 (4.0-12.2) | 0.81 (0.60-1.10) |
|  |  | ≥30y | 183 (14.6%) | 4.4 (3.7-5.1) | 1.28 (0.98-1.69) |
|  |  | Trend, χ^2^ (*p* value) |  |  | 0.53 (0.47) |
| Smoking and alcohol drinking status | | |  |  |  |
|  | Never smoker and never drinker | | 746 (59.7%) | 5.4 (4.4-6.6) | 1 (Reference) |
|  | Current smoker and never drinker | | 204 (16.3%) | 5.2 (3.5-7.5) | 1.00 (0.75-1.25) |
|  | Current drinker and never smoker | | 47 (3.8%) | 5.0 (2.7-12.1) | 1.14 (0.67-1.93) |
|  | Current smoker and current drinker | | 253 (20.2%) | 4.5 (3.7-5.6) | 1.21 (0.93-1.58) |
